# Mind the Gap: Investigating Toddlers’ Sensitivity to Contact Relations in Predictive Events

**DOI:** 10.1371/journal.pone.0034061

**Published:** 2012-04-13

**Authors:** Paul Muentener, Elizabeth Bonawitz, Alexandra Horowitz, Laura Schulz

**Affiliations:** 1 Department of Brain & Cognitive Sciences, Massachusetts Institute of Technology, Cambridge, Massachusetts, United States of America; 2 Department of Psychology, University of California, Berkeley, California, United States of America; 3 Department of Psychology, Stanford University, Palo Alto, California, United States of America; Royal Holloway, University of London, United Kingdom

## Abstract

Toddlers readily learn predictive relations between events (e.g., that event A predicts event B). However, they intervene on A to try to *cause* B only in a few contexts: When a dispositional agent initiates the event or when the event is described with causal language. The current studies look at whether toddlers’ failures are due merely to the difficulty of initiating interventions or to more general constraints on the kinds of events they represent as causal. Toddlers saw a block slide towards a base, but an occluder prevented them from seeing whether the block contacted the base; after the block disappeared behind the occluder, a toy connected to the base did or did not activate. We hypothesized that if toddlers construed the events as causal, they would be sensitive to the contact relations between the participants in the predictive event. In Experiment 1, the block either moved spontaneously (no dispositional agent) or emerged already in motion (a dispositional agent was potentially present). Toddlers were sensitive to the contact relations only when a dispositional agent was potentially present. Experiment 2 confirmed that toddlers inferred a hidden agent was present when the block emerged in motion. In Experiment 3, the block moved spontaneously, but the events were described either with non-causal (“here’s my block”) or causal (“the block can make it go”) language. Toddlers were sensitive to the contact relations only when given causal language. These findings suggest that dispositional agency and causal language facilitate toddlers’ ability to represent causal relationships.

## Introduction

Human adults recognize that events that predict each other sometimes cause each other. This allows us to generate novel interventions, distinguish spurious associations from genuine causes, represent physical relationships among events, and engage in effective exploration [1]–[5]. However, many researchers have speculated that this recognition might emerge relatively late in both phylogeny and ontogeny [2], [5]–[8]. In this study, we use a looking-time method to explore whether toddlers have access to a concept of causation that integrates an understanding of predictive relationships, interventions and physical relations among events.

Recent research suggests that this integrated understanding may be absent in early childhood [6]. In particular, although preschoolers readily move from observing predictive relationships among physical events to trying causal interventions, toddlers do not. In a study upon which the current work is based [6], children were familiarized to a two-part predictive event in which (1) a block moved across a stage and contacted a base, and (2) a spinning toy airplane, connected by a visible wire to the base, immediately activated. Preschoolers (mean: 47 months) and toddlers (mean: 24 months) were equally successful at learning the predictive relationship: in a catch trial, in which the toy did not activate, virtually all the children spontaneously looked to the toy. However, when asked to make the toy go, almost all the preschoolers pushed the block towards the base and looked to the toy; none of the toddlers did so. That is, no toddler spontaneously initiated the action, and when prompted to perform the action, all of the toddlers pushed the block to the base but none predictively looked to the toy.

Two factors appeared to affect toddlers’ ability to move from prediction to intervention: the presence of a dispositional agent (i.e., an agent capable of intentional action) and the inclusion of causal language. If instead of the block moving by itself during the familiarization phase, the experimenter pushed the block into the base, then toddlers performed the action themselves and anticipated the outcome. If the events were described with causal language (e.g., “The block can make it go”), toddlers’ performance improved; neutral language (“Look at the block!”) did not improve performance.

These findings suggest that toddlers may not understand that predictive relations can be potential causal events. Although there are developmental changes in children’s causal knowledge [9]–[11], a wealth of recent research has stressed the sophistication of children’s causal reasoning abilities [12]–[14]. Critically however, the causal events in such studies are almost always initiated by dispositional agents (puppets or people) and/or described with causal language (e.g., “blickets make the toy go”). Exceptions to this trend are Michottean launching events, in which one object strikes another object and immediately sets it in motion. However, some researchers have suggested that infants’ causal perception of launching events may be modular and distinct from other forms of causal inference [15]. Thus, little is known about whether children spontaneously recognize the possibility that non-agentive predictive relations are causal – a possibility recognized by 4-year-old children [6] and arguably adults. By contrast, the importance of dispositional agency to infants’ causal representations has been widely documented. Infants represent dispositional agents, but not objects, as potential causes of both object motion and change of state events [16]–[18]. Arguably then, in the absence of dispositional agency, toddlers, like infants, might fail to represent predictive relations as potentially causal.

Alternatively, toddlers may represent predictive relations as potential causal relations but have difficulty initiating causally relevant actions for certain kinds of events. Researchers have suggested that intentional action might, *in general*, lag behind predictive looking either because the demands of planning and executing motor responses interfere with children’s ability to access task-relevant information [19]–[21], or because stronger representations might be necessary for acting than for looking [22]. Although there are important theoretical distinctions between these claims, they are united in suggesting that a gap between children’s ability to make successful predictions and their ability to perform effective actions might reflect changes not in children’s conceptual understanding but in their ability to manifest their knowledge under complex task demands. If so, any additional information that strengthens the representation of a causal relationship might boost performance.

By assessing toddlers’ reasoning about predictive relations independent of their ability to initiate actions, we can learn whether dispositional agency and causal language merely facilitate children’s ability to move from prediction to intervention, or whether these factors affect children’s underlying conceptual representations of predictive relations. In order to distinguish these two accounts, we investigate children’s sensitivity to spatial relations between causal agents and patients in physical events using a looking time study. Of course spatial contact is neither necessary nor sufficient for causality. As philosophers have pointed out, if mere transmission through contact were sufficient for causal inference, the transfer of chalk from a billiard stick to a billiard ball would be as probable a cause of the ball’s subsequent motion as the transfer of force [23]. Moreover, psychological causal events, including chasing and fleeing events, occur without physical contact [24], [25]. Nonetheless, prior research suggests that infants’ expectations about spatial contact in the domain of physical outcomes vary depending on whether an outcome does or does not occur. In events that adults typically recognize as physical causal interactions, research has shown that infants (1) expect outcomes to occur on contact and (2) expect outcomes not to occur at a distance [16], [26]–[30]. For example, 7.5-month-old infants have been shown to expect an object to move upon contact from another object, and not to move if an object stops short of contacting it [27]. Similarly, infants who have seen a hand move behind an occluder and a box break apart expect the hand to have contacted the box if it breaks and expect the hand to have stopped short of contact if the box does not break; moreover, they do not have these expectations if the candidate cause is not a dispositional agent (e.g., is a train rather than a hand) [16]. Following the logic of these studies, in the current study we look at whether toddlers represent predictive relations in accord with principles of physical contact causality.

Using an occluded causal inference paradigm [16], [26], [29], we show toddlers a block that slides towards a base; a toy connected to the base either does or does not activate. An occluder prevents children from seeing whether the block contacts the base. On test, we remove the occluder and measure looking time. If toddlers’ failure to intervene in Bonawitz and colleagues [6] is due only to the difficulty involved in initiating motor responses, then children should be sensitive to the spatial relations in all the events. By contrast, if toddlers require dispositional agency or causal language to represent the causal nature of predictive events, then they should be sensitive to the spatial relations as a function of the outcome in the presence of these cues, but not in their absence. This paradigm thus allows us to investigate whether dispositional agency and causal language are factors that merely *strengthen* existing causal representations and support successful interventions or whether these factors are required for initially representing such events as causal. In Experiments 1 and 2, we investigate the influence of dispositional agency; in Experiment 3, we investigate the influence of causal language.

## Results and Discussion

In Experiment 1, we investigated the primary cue shown to facilitate toddlers’ ability to represent predictive relations as potential causal relations: having the event initiated by a dispositional agent. We predicted that toddlers would be sensitive to spatial relations for events initiated by a dispositional agent, but not for otherwise identical events that do not involve dispositional agents.

Unlike prior research that compared toddlers’ causal reasoning about events involving dispositional agents to events involving objects, we used an *inferred* dispositional agent rather than a visible dispositional agent. In this we were inspired by previous research [17], [18] suggesting that infants posit hidden agents when an object emerges in motion. If for instance, a beanbag emerges from the right side of a stage, 7-month-olds look longer when a previously hidden hand is revealed on the left side of the stage than the right. We used an inferred dispositional agent to preclude the possibility that toddler’s looking times could simply be driven by attention to the agent itself (i.e., infants might look longer at events involving hands than events that do not). Thus, we compared two closely matched conditions: one in which a block began to move spontaneously (the Spontaneous condition) and one in which a block emerged from off-stage already in motion (the Inferred Agent condition). If toddlers represent hidden agents when objects emerge in motion, and represent agent-initiated but not spontaneously occurring events as causal, they should be sensitive to spatial relations in the Inferred Agent condition but not the Spontaneous condition.

**Figure 1:**
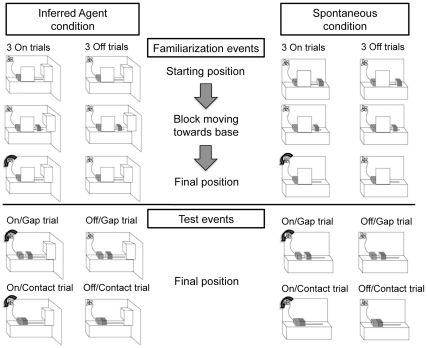
Procedure used in Experiments 1–3. In Experiment 1, toddlers were assigned to either the Inferred Agent or Spontaneous condition. They viewed 6 familiarization trials (3 On, 3 Off, in alternation) in which a block emerged from behind the right side barrier (row 1), traveled towards the base on the left side of the stage (row 2), and disappeared behind the screen (row 3). The beginning of the test events was identical to the familiarization events (rows 1–2). Following the disappearance of the block, the experimenter removed the screen to reveal the block in contact or at a distance from the base, and the toy either on or off. Experiment 2 used the same materials depicted in the Inferred Agent condition; the block began moving either from onstage (Spontaneous condition) or from behind the right-side barrier (Inferred Agent condition). Instead of the test events depicted, the right-side barrier was removed on test to reveal a hand behind the barrier. Experiment 3 used the Familiarization and Test procedures depicted in the Spontaneous condition and the events were described with either causal or non-causal language.

Toddlers first viewed 6 familiarization events (3 *On* trials, 3 *Off* trials, in an alternating presentation) in which a block traveled across a stage towards a base block, disappearing behind an occluder (see Figure 1). Toddlers saw the block start its motion from rest on the stage (Spontaneous condition) or saw it emerge from behind an initial barrier (Inferred Agent condition). In *On* trials, following the block’s disappearance, a toy plane, connected to the base via a wire began to spin. In *Off* trials, the block travelled behind the occluder, but the airplane did not spin. Following the familiarization phase, toddlers viewed a single test trial in which the block moved towards the base, disappearing behind the occluder. The toy then activated during the test event (Toy On conditions) or did not (Toy Off conditions). Following the toys’ activation (or failure to activate), the occluder was removed to reveal the block either touching (Contact) or at a distance (Gap) from the base. We measured looking time to the test event display (Toy On/Contact, Toy Off/Contact, Toy On/Gap, or Toy Off/Gap) until the toddler looked away for 2 consecutive seconds.

**Figure 2:**
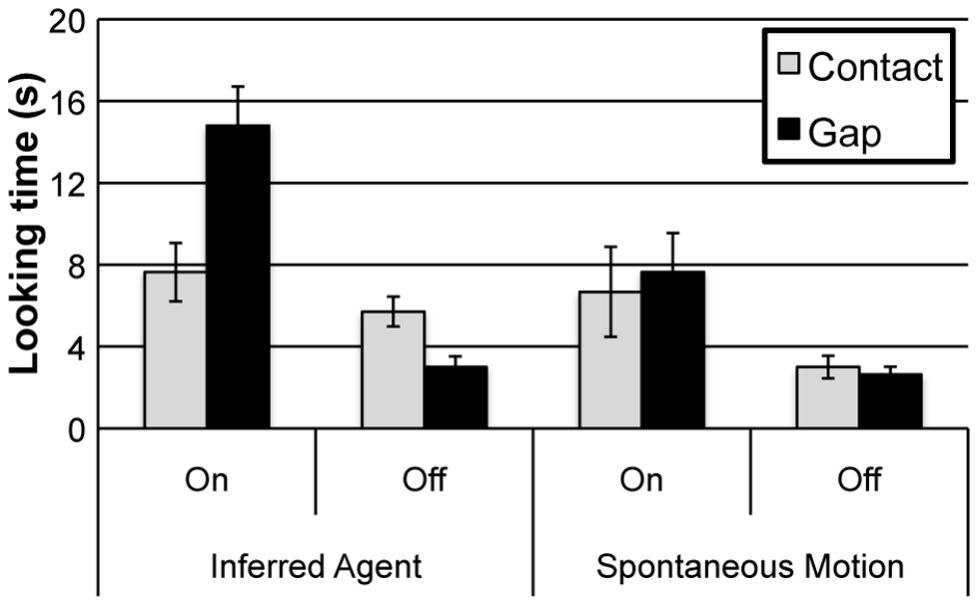
Results from Experiment 1. Looking time (±1 SD) to the final test events in Experiment 1.

To evaluate toddlers’ looking time to the test events (see Figure 2), we conducted an analysis of variance (ANOVA) with Agency (Inferred Agent vs. Spontaneous), Activation (Toy On vs. Toy Off), and Spatial Relation (Contact vs. Gap) as between-subjects factors. This analysis yielded a main effect of Activation, F(1, 88)  =  32.69, *p*<.0001. Toddlers looked longer at the test event when the airplane moved (9.19 s) than when it did not (3.58 s). There was also a 2-way interaction between Activation and Spatial Relation, F(1, 88)  =  8.88, *p* = .004 which was qualified by a 3-way interaction between Agency, Activation, and Spatial Relation, F(1, 88)  =  4.19, *p* = .044. There were no other main effects or interactions.

We conducted separate ANOVAs in each condition to follow-up this analysis. In the Inferred Agent condition, there was a main effect of Activation; F(1, 44)  =  28.95, *p*<.0001. Toddlers looked longer when the airplane moved (11.22 s) than when it did not (4.35 s). There was also an interaction between Activation and Spatial Relation, F(1, 44)  =  14.94, *p*<.001. This interaction reflected the fact that toddlers looked longer at the gap event when the airplane moved, *t*(22)  =  2.99, *p* = .007, but longer at the contact event when the airplane did not move, *t*(22)  =  3.00, *p* = .007.

A different pattern emerged in the Spontaneous condition. There was a main effect of Activation, *F*(1, 44)  =  8.53, *p* = .005. Toddlers looked longer when the airplane moved (7.2 s) than when the airplane did not (2.8 s). No other main effects or interactions approached significance. In the Spontaneous condition, toddlers did not discriminate among the test events.

Results from Experiment 1 suggest that only toddlers in the Inferred Agent condition represented the block as the cause of the airplane’s motion. These children looked longest when the test event violated contact causality: (1) when the block stopped short of the base but the toy activated or (2) when the block contacted the base but the toy did not activate. By contrast, when the block moved spontaneously, toddlers did not differentiate the test events. Thus, strikingly, merely occluding the onset of the block’s motion allowed toddlers to make predictions about contact causality that they failed to make when the onset of motion was visible. These results are consistent with the hypothesis that dispositional agency facilitates toddlers’ ability to represent predictive relations as causal.

However, while consistent with this hypothesis, we have no *positive* evidence that toddlers’ success in the Inferred Agent condition in Experiment 1 was due to inferring the presence of a hidden agent. To test this, we presented the same familiarization events in Experiment 2 as in Experiment 1 but at test, rather than reveal the spatial relation between the block and the base, we removed the barrier on the right of the stage to reveal a person’s hand; the airplane always activated during the test event. Following the logic of Saxe and colleagues [17], [18], if toddlers infer a hidden agent only when the block emerges in motion, then toddlers in the Spontaneous condition should look longer at the hand than those in the Inferred Agent condition.

Toddlers’ looking times supported this prediction; toddlers inferred that there was a hidden dispositional agent when the block emerged in motion but not when it moved spontaneously. An analysis of toddlers’ looking time to the test event revealed that toddlers looked significantly longer in the Spontaneous (17.62 s) than the Inferred Agent condition (9.96 s), *t(22)*  =  3.43, *p* = .002. This is consistent with the hypothesis that toddlers represented the events causally in the Inferred Agent condition of Experiment 1 because they believed that a dispositional agent initiated the events.

Why did toddlers fail to make differential predictions in the Spontaneous condition? We have suggested that toddlers do not readily represent objects as causes; they thus failed to represent the non-agent event causally. However, the spontaneous movement of the block itself violated the expectation that physical objects move only when they are contacted [27]. Thus, the initial spontaneous movement of the block might have confused the toddlers and disrupted any further expectations.

Arguing against this possibility, note that there was no difference between conditions in the number of toddlers who met the inclusion criteria (i.e., who predictively looked to the plane during the familiarization phase). Nor was there any difference between conditions in toddlers’ overall looking times. This suggests that children did not find the spontaneous movement of the block particularly disruptive.

However, if as we have suggested, it is the absence of a dispositional agent rather than the presence of spontaneous movement that interferes with children’s expectations of contact causality, then even in the face of spontaneous movement children should represent contact causality given other cues to the causal relationship. Previous research [6] suggests that causal language acts as such a cue. When spontaneously occurring events are described causally, toddlers intervene and anticipate the target outcome. Causal language might help extend children’s causal representations for either of two (not mutually exclusive) reasons. First, causal language testifies that an observed relationship is indeed an instance of direct causation. Second, the fact that the same language is used to describe predictive relations and agent interventions (e.g., “The block can *make it go*.”; “Can you *make it go*?”) might highlight the common underlying structure [6].

In Experiment 3 we looked at whether causal language similarly supports toddler’s sensitivity to contact causality. In the *Causal Language* conditions, the experimenter drew children’s attention to the stage at the onset of each familiarization trial by saying, “The block can make it go.” The *Non-causal Language* condition was identical except the experimenter said, “Here’s my block.” If causal language supports toddlers’ sensitivity to contact causality, then this would suggest both that spontaneous movement is not itself an obstacle to toddlers’ causal representations, and that causal language affects children’s representations of events, not merely their ability to engage in causally relevant actions. Alternatively, if toddlers are simply confused by the spontaneous motion of the block, which then interferes with their subsequent representations of the event, then they should not be sensitive to contact relations even in the presence of causal language.

**Figure 3:**
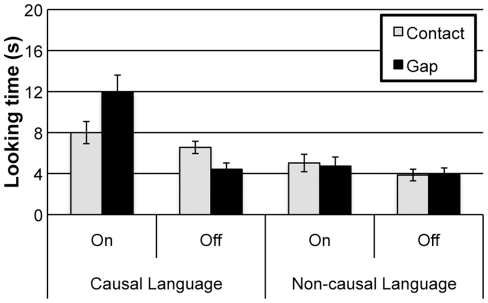
Results from Experiment 3. Looking time (±1 SD) to the final test events in Experiment 3.

An analysis of toddlers’ looking time to the final test event (see Figure 3) revealed that they were sensitive to contact causality only in the Causal Language condition. We conducted an ANOVA with Language (Causal vs. Non-Causal), Activation (Toy On vs. Toy Off), and Spatial Relation (Contact vs. Gap) as between-subjects factors. The analysis yielded a significant main effect of Language, F(1, 120)  =  26.99, *p*<.0001 and a main effect of Activation, F(1, 120)  =  18.00, *p*<.0001. Toddlers looked longer in the Causal language (7.72 s) than the Non-causal language conditions (4.40 s) and looked longer when the airplane moved (7.43 s) than when it did not (4.70 s). Second, this analysis yielded significant 2-way interactions between Language and Activation, F(1, 120)  =  7.52, *p* = .007, and between Spatial Relation and Activation, F(1, 120)  =  4.83, *p* = .03, which were qualified by a 3-way interaction between the Language, Activation, and Spatial Relation, F(1, 120)  =  6.44, *p* = .012.

To follow-up this analysis, we conducted separate ANOVAs on toddlers’ looking time to the test event in each condition, with Activation and Spatial Relation as between-subject factors. In the Causal language condition, there was a significant main effect of Activation, F(1, 60)  =  17.85, *p*<.0001. Toddlers looked longer when the airplane moved (9.97 s) than when it did not (5.48 s). There was also a significant interaction between Activation and Spatial Relation, F(1, 60)  =  8.21, *p* = .006. This interaction reflected the fact that toddlers looked longer at the gap event when the airplane moved, *t*(30)  =  2.02, *p* = .05, but looked longer at the contact event when the airplane did not move, *t*(30)  =  2.50, *p* = .018.

A different pattern emerged in the Non-causal language condition. The three-way ANOVA yielded no significant main effects or interactions (all *p*s>.05). In the absence of causal language, toddlers did not discriminate among the test events.

The results from Experiment 3 suggest that only toddlers in the Causal Language condition represented the predictive relationship as a causal relationship. In that condition, toddlers looked longest (1) when the block had not made contact with the base when the airplane activated and (2) when the block had made contact with the base when the airplane failed to activate. These results additionally suggest that spontaneous movement does not prevent toddlers from forming causal representations; when spontaneously occurring events were described with causal language, toddlers were sensitive to the contact relations. Together with previous work [6], the results of Experiments 1 and 3 suggest that in the absence of a dispositional agent and causal language toddlers do not merely have difficulty initiating causal interventions, but they do not even have expectations about contact between the potential cause and effect.

Thus, the current findings suggest that toddlers are sensitive to spatial relations as a function of the outcome only when they can infer the presence of a dispositional agent (Experiment 1) or when events are described with causal language (Experiment 3). Prior research [6] showed that dispositional agency and causal language facilitate toddlers’ ability to spontaneously intervene on predictive relations. Together with the current findings, it appears that dispositional agency and causal language support children’s ability to represent predictive relations among novel events as causal relations.

The conclusions from the current study rest upon an assumption that toddlers’ differential expectation of spatial relations can be taken as evidence for causal representations. As reviewed in the Introduction, we believe that sensitivity to contact relations *as a function of the outcome* suggests that children have interpreted an event causally. In the current study, toddlers did not simply discriminate contact events from gap events in the Inferred Agent and Causal Language conditions; their looking patterns differed depending on the toy’s activation. If the outcome occurred, toddlers looked longer at gap events; if the outcome did not occur, looked longer at contact events. Infants tend to show this pattern of looking only for physical events in which adults typically represent causally. This does not imply that children expect all causal outcomes to require contact; prior research suggests that children may suspend contact constraints for non-physical, social interactions such as chasing/fleeing [24], [25]. Moreover, we do not suggest that young children would be unable to learn causal relationships between events that do not involve contact; when a dispositional agent initiates the events, children readily learn such relations (e.g., between flipping a switch and a light turning on) [6]. However, this study adds to the evidence that, for those events that adults represent causally, infant have differential expectations about contact relations as a function of event outcomes.

In the current study, we assumed that toddlers would represent the block and the base as objects (i.e., rather than agents). Arguably however, and contrary to our intended manipulation, toddlers might have represented the block as a dispositional agent in the Spontaneous and language conditions because the block engaged in self-propelled motion. There are several reasons however, to believe that this was not the case. First, previous research suggests that self-propelled motion is not, in and of itself, a sufficient cue for infants and toddlers to attribute agency [24], [25], [31]–[35]; children require additional cues (e.g., contingent responding, non-Newtonian motion). Second, if, toddlers had attributed animacy to the block, they should have succeeded in all conditions of the current study, as an agent (visible or inferred) would then have been involved in all of the predictive events. Third, in previous work using a very similar paradigm [6] toddlers had ample opportunity to interact with the block and toddlers engaged only in object-directed play (e.g., banging the block) never in agent-directed play (e.g., talking to the block). Finally, if toddlers had treated the block as an agent in the Spontaneous and Language conditions than in the Inferred Agent condition, one might have expected them to look longer at the block overall in those conditions; however, we found no overall differences in looking time across conditions.

Thus we believe that the results of the current study are consistent with other work showing that infants accept dispositional agents, but not objects, as candidate causes of physical motion [36] and change of state events [16]. Michottian launching events remain an important exception; infants as young as 6-months distinguish causal agents and causal patients in launching events, even though no dispositional agents are present [37]. However, such “perceptual causality” depends on the precise spatio-temporal properties of the events, suggesting it might be encapsulated from other kinds of causal reasoning [15], [37]–[41]. The current findings suggest that, outside of arguably modular processes, children might not represent the causal structure of non-agentive events until relatively late in development.

If so, language may play an important role in extending children’s causal representations. Simply by testifying that a novel event is indeed causal, language might broaden the range of events that toddlers recognize as instances of causation. Additionally, the use of common causal language across superficially distinct contexts might help children integrate initially distinct representations (e.g., of predictive relations, spatiotemporal relations, and the outcome of interventions) into an adult-like notion of causality [6], [42]. Further research is necessary to better understand the interactions between language and causal reasoning and to establish precisely which aspects of causal language affect children’s causal representations.

Finally, we note that the use of infant looking time as a measure of conceptual understanding has been subject to debate [43], [44]. This study establishes a convergence between looking time measures (used here) and the action measures used in closely matched previous work [6]. This convergence may help validate sensitivity to contact causality as an index of causal understanding in infancy research [16], [26], [27], [29], [30].

These findings highlight the importance of dispositional agency and causal language in the development of causal reasoning. Although further research is needed to uncover the trajectory of causal representations in early childhood and the precise role of causal language in supporting these representations, the current study helps fill the gap between research on infants’ restricted causal representations and the sophisticated causal reasoning of later childhood.

## Materials and Methods

### Ethics Statement

The Massachusetts Institute of Technology Institutional Review Board approved the procedures for all research described in this paper. We obtained written consent from the participants’ parents.

### Participants

Two-hundred-forty-eight toddlers (mean: 24.1 months, range –18–30 months) were recruited at a Children’s Museum. An additional 20 toddlers were recruited but not included in the final sample due to: inability to complete the session (n  =  7), parental interference (n  =  3), or failure to predictively look during the familiarization trials (n  =  10). In Experiment 1, twelve toddlers were assigned to each of eight conditions crossing three factors: Agency (Inferred Agent or Spontaneous), Activation (Toy On or Toy Off), and Spatial Relation (Contact or Gap). In Experiment 2, toddlers were assigned to an Inferred Agent or a Spontaneous condition. In Experiment 3, sixteen toddlers were assigned to each of eight conditions crossing three factors: Language (Causal or Non-causal), Activation (Toy On or Toy Off), and Spatial Relation (Contact or Gap). There were no age differences across conditions within each experiment.

### Materials

All events occurred on a white stage (60 cm^2^×50 cm^2^) that hid a confederate from view. A barrier was positioned to the far right of the stage (See Figure 1). An orange block (the base) and a purple block (both 6 cm^3^) were on opposite ends of the stage. The purple block was attached to a stick extending through the floor of the stage, allowing the hidden confederate to surreptitiously move the block across the stage to the base. A toy airplane, visibly attached to the base by an orange wire, was located on the back stage wall. During familiarization, a screen (22×28 cm) occluded the spatial relationship between the block and base.

### Procedures

#### Experiment 1

The block began at the far right of the stage in the Spontaneous condition and behind the right side barrier in the Inferred Agent condition (See Figure 1). The experimenter drew the toddlers’ attention to the stage saying, “Watch my show.” Toddlers viewed an *On trial* and then an *Off trial*. In the *On trial*, the block moved towards the base and disappeared behind the screen. Once the block disappeared, the airplane began to spin. At the end of the trial the stage was covered by a curtain and the scene was reset. The *Off* trials were identical, except that the airplane did not spin. The experimenter ended the trial after the airplane spun for 3 s (*On* trial) or (*Off* trial) after the toddler predictively looked towards the airplane or 3 s, whichever came first. This procedure was repeated twice, for a total of 6 familiarization trials. In order to proceed to the test phase, toddlers had to predictively look to the airplane on at least two *Off* trials. For all experiments, there were no significant differences across conditions in the number of toddlers who were dropped from subsequent analysis because they failed to predictively look during the *Off* familiarization trials.

The start of each test event was identical to the familiarization: the block moved towards the base, disappearing behind the screen. Toddlers either saw events in which the airplane activated during the test event (Toy On conditions) or did not (Toy Off conditions). The experimenter then said, “Look at this!” and removed the screen, revealing the block either touching (Contact conditions) or at a distance (Gap conditions) from the base.

#### Experiment 2

The familiarization phase in Experiment 2 was similar to Experiment 1, with the following exception. In the Inferred Agent condition, the block emerged from off-stage already in motion. In the Spontaneous condition, the block was adjacent to the barrier and began moving spontaneously. Toddlers viewed 6 familiarization events identical to those in Experiment 1. Following familiarization, all toddlers viewed the same test event. The block moved towards the base, disappearing behind the occluder. The experimenter said, “Look at this!” and lowered the far right barrier, revealing a hand at rest, palm facing the block.

#### Experiment 3

The procedure mirrored the Spontaneous condition in Experiment 1, except that at the start of each familiarization trial the experimenter used either causal or non-causal language to draw children’s attention to the stage. In the Causal Language conditions, the experimenter said, “The block can make it go.” In the Non-causal Language condition, the experimenter said, “Here’s my block.” Although the conditions differed in the reference to “it” (i.e., the toy plane), this difference did not seem to affect children’s ability to encode the relationship given that toddlers were equally good at learning the predictive relationship between the block and the toy in both conditions.

### Coding

The experimenter ended each familiarization trial when he judged that the child looked away for 2 consecutive seconds. The experimenter viewed the toddlers’ looking towards the entire stage by looking at the video camera screen that recorded the toddlers’ looking. In a few instances, the experimenter misjudged the 2-second look-away criterion and ended the test event early, before the child had actually looked away for 2 consecutive seconds. The data from these participants were removed and subsequently replaced with data from new participants. For all experiments, there were no significant differences across conditions in the number of toddlers removed and replaced.

Following data collection, looking times were coded from video, with the coder blind to the test event. A third of the clips from each Experiment were reliability coded; inter-rater reliability was high throughout, r^2^>.9. The first coder’s data were used for all analyses.

## References

[1] Glymour C, Spirtes P, Scheines R (2001). Causation, prediction and search..

[2] Gopnik A, Glymour C, Sobel D, Schulz L, Kushnir T (2004). A theory of causal learning in children: Causal maps and Bayes nets.. Psychol Review.

[3] Pearl J (2000). Causality: Models, reasoning, and inference..

[4] Schulz L, Gopnik A, Glymour C (2007). Preschool children learn about causal structure from conditional interventions.. Developmental Sci.

[5] Woodward J, Gopnik A, Schulz L (2007). Interventionist theories of causation in psychological perspective.. Causal learning.

[6] Bonawitz E, Ferranti D, Saxe R, Gopnik A, Meltzoff A (2010). Just do it? Toddlers’ ability to integrate prediction and action.. Cognition.

[7] Meltzoff A (1995). Understanding the intentions of others: re-enactment of intended acts by 18-month-old children.. Dev Psychol.

[8] Meltzoff A, Blumenthal E (2007). Causal understanding and imitation: Effect monitoring in infants..

[9] Bullock M, Gelman R, Baillargeon R, Friedman W (1982). The development of causal reasoning.. The developmental psychology of time.

[10] Schulz T (1982). Rules of causal attribution.. Monogr Soc Res Child, 194,.

[11] Sperber D, Premack D, Premack A (1995). Causal Cognition: A multidisciplinary debate..

[12] Gopnik A, Schulz L (2004). Mechanisms of theory-formation in young children.. Trends Cogn Sci.

[13] Schulz L, Kushnir T, Gopnik A, Gopnik A, Schulz L (2007). Learning from doing: Intervention and causal inference.. Causal learning.

[14] Gopnik A, Schulz L (2007). Causal learning..

[15] Scholl B, Tremoulet P (2000). Perceptual causality and animacy.. Trends Cogn Sci.

[16] Muentener P, Carey S (2010). Infants’ causal representations of state change events.. Cognitive Psychol.

[17] Saxe R, Tenenbaum J, Carey S (2005). Secret agents: Inferences about hidden causes by 10- and 12-month-old infants.. Psychol Sci.

[18] Saxe R, Tzelnic T, Carey S (2007). Knowing who dunnit: Infants identify the causal agent in an unseen causal interaction.. Dev Psychol.

[19] Baillargeon R, Graber M, Devos J, Black J (1990). Why do young infants fail to search for hidden objects?. Cognition.

[20] Diamond A, Goldman-Rakic P (1989). Comparison of human infants and rhesus monkeys on Piaget's AB task: evidence for dependence on dorsolateral prefrontal cortex.. Exp Brain Res.

[21] Thelen E, Smith L (1994). A Dynamic Systems Approach to the Development of Cognition and Action..

[22] Munakata Y (2001). Graded representations in behavioral dissociations.. Trends Cogn Sci.

[23] Woodward J (2003). Making things happen: A theory of causal explanation..

[24] Schlottmann A, Surian L (1999). Do 9-month-olds perceive causation-at-a-distance?. Perception.

[25] Schlottmann A, Surian L, Ray E (2009). Causal perception of action-and-reaction sequences in 8- to 10-months-old infants.. J Exp Child Psychol.

[26] Ball W (1973). The perception of causality in the infant..

[27] Kotovsky L, Baillargeon R (2000). Reasoning about collision events involving inert objects in 7.5-month-old infants.. Developmental Sci.

[28] Leslie A (1984). Spatiotemporal continuity and the perception of causality in infants.. Perception.

[29] Woodward A, Phillips A, Spelke E (1993). Infants’ expectations about the motion of animate versus inanimate objects..

[30] Luo Y, Kaufman L, Baillargeon R (2009). Young infants’ reasoning about physical events involving self- and nonself-propelled objects.. Cognitive Psychol.

[31] Shimizu Y, Johnson S (2004). Infants’ attribution of a goal to a morphologically unfamiliar agent.. Developmental Sci.

[32] Csibra G, Gergely G, Biro S, Koos O, Brockbank M (1999). Goal attribution without agency cues: the perception of ‘pure reason’ in infancy.. Cognition.

[33] Johnson S, Slaughter V, Carey S (1998). Whose gaze will infants follow? The elicitation of gaze following in 12-month-olds.. Developmental Sci.

[34] Movellan J, Watson J (2002). The development of gaze following as a Bayesian systems identification problem..

[35] Schlottmann A, Ray E (2010). Goal attribution to schematic animals: Do 6-months-olds perceive biological motion as animate?. Developmental Sci.

[36] Saxe R, Carey S (2006). The origin of the idea of cause: Critical reflections on Michotte’s theory with evidence from infancy.. Acta Psychol.

[37] Leslie A, Keeble S (1987). Do six-month-old infants perceive causality?. Cognition.

[38] Oakes L, Cohen L (1990). Infant perception of a causal event.. Cognitive Dev.

[39] Michotte A (1947). The perception of causality..

[40] Blakemore S, Fonlupt P, Pachot M, Darmon C, Boyer P (2001). How the brain perceives causality: An event-related fMRI study.. Neurological Report.

[41] Schlottmann A (2000). Is perception of causality modular?. Trends Cognit Sci.

[42] Muentener P, Schulz L (2012). What does and doesn’t go without saying: Communication, induction, and exploration.. Language Learning & Development.

[43] Haith M (1998). Who put the cog in infant cognition? Is rich interpretation too costly?. Infant Behav Dev.

[44] Kidd C, Piantadosi S, Aslin R (2010). The Goldilocks Effect: Infants’ preference for visual stimuli that are neither too predictable nor too surprising..

